# Creation and verification of a predictive nomogram model for the incidence of social isolation among China’s older population

**DOI:** 10.3389/fpubh.2025.1571509

**Published:** 2025-07-16

**Authors:** Mei You, Yuan Ding, Zixuan Wei, Nannan Han, Annuo Liu

**Affiliations:** ^1^Department of Anesthesiology, The First Affiliated Hospital of Anhui Medical University, Hefei, China; ^2^School of Nursing, Anhui Medical University, Hefei, China

**Keywords:** social isolation, older adult population, nomogram model, risk factors, predictive accuracy

## Abstract

**Objectives:**

To explore the risk factors associated with social isolation among the older adult in China, develop a nomogram model to forecast the risk, and evaluate its predictive accuracy.

**Methods:**

An investigation was conducted into the demographic, socioeconomic, health, and health behavior aspects of the older adult population. Using logistic regression and backward stepwise analysis, a nomogram model was constructed to predict the risk of social isolation by screening independent risk factors.

**Results:**

Social isolation was prevalent in 42.1% of Chinese older adults. Nomogram prediction models were created for the five screened variables, which included type of residence, health self-assessment, disability, depression, and sedentary hours. The nomogram model had an AUC of 0.734 (95%CI: 0.701–0.767) in the training cohort, and an AUC of 0.653 (95%CI: 0.580–0.725) in the validation cohort. The Hosmer-Lemeshow goodness-of-fit test revealed that there was a good fit (*p* > 0.05). DCA results showed that clinical intervention had a high net benefit in the older adult when the threshold probability was 20–85% for the training cohort and 30–65% for the control cohort.

**Conclusion:**

Social isolation is a common issue for the older adult population in China. The prediction model using a nomogram for the older adult can efficiently detect and screen high-risk individuals for social isolation, forecasting its occurrence. The proposed nomogram may serve as a preliminary screening tool for social isolation risk but requires further optimization to improve accuracy. Future research should incorporate additional predictors or advanced modeling techniques to enhance clinical utility.

## 1 Introduction

In 2022, the Party Group of the China Health and Wellness Commission highlighted in ‘Writing a New Chapter on Population Work in the New Era’ that China is projected to undergo major population aging by 2035 (1). Several studies have pointed out that the social network of older adults, as an important social resource, is closely related to the health and quality of life of older adults (2–4). As they progress into old age, older adults often see a decline in social functions, reduced social engagement, and less developed social networks due to changes in family roles, social responsibilities, and physical conditions, which can lead to older adults becoming at high risk for social isolation.

Social isolation refers to either an active or passive withdrawal from society, where personal interactions, participation in activities, and social connections are all severed or separated (5). In some developed nations like the United States, Germany, and Switzerland, the incidence of older adult social isolation ranges from 11.9 to 28% (6–8); in some developing nations like China, India, and Iran, it ranges from 19.7 to 41.3% (9–11); Among these countries, China has a relatively high older adult social isolation rate of 41.3% (11). This may be related to the accelerating process of urbanization in China and the prominence of empty nesting and living alone among the older adult (12). With the rapid aging of Chinese society, addressing the social isolation of the older adult in China has become crucial for promoting active aging.

In addition to the commonality of social isolation, the health promotion activities of seniors can be influenced by their social isolation status, possibly causing more severe detrimental health effects. Several studies have reported that older persons who experience social isolation have a 29% higher risk of death (13), a 50% higher chance of dementia, a 29% higher risk of coronary heart disease (14), a 32% higher risk of stroke (15), and a 26% higher incidence of hospital readmission (16). Additionally, social isolation has been identified as a factor associated with suicidal thoughts or ideation in older adults (17). Given this, it becomes particularly important to identify older adults at high risk of social isolation before it occurs.

According to previous studies, social isolation in older adults is affected by a variety of factors, including gender, age, marital status, education, economic status, chronic illness, unhealthy lifestyle choices (like smoking and inactivity), physical dysfunction, cognitive impairment, and depression (10, 18–21). According to recent research, individual health risk behaviors tend to occur together in clusters (22), and individuals with multiple health risk behaviors face more health challenges than those with a single health risk behavior (23). As a result, when developing predictive models of social isolation in older adults, the impact of multiple health risk behaviors acting concurrently on individuals should be considered in order to assist healthcare providers in more accurately identifying those at high risk of social isolation at an early stage.

This study explores the factors influencing the occurrence of social isolation among community-dwelling older adults in terms of individual characteristics and environmental characteristics based on social cognitive theory (24). Social cognitive theory can be used to explain predictive ability, and foreign scholars have applied it to motor behavior and explained the predictive value of the theory (25). In recent years, domestic researchers have applied its theoretical results to various behavioral fields, focusing on the use of data analysis and quantitative methods to study the influencing factors and mechanisms of a particular behavior, which can provide a good theoretical basis for the analysis of the influencing factors of social isolation of the older adult in the preliminary stage of this study.

The nomogram is a visual statistical model that calculates the risk score for the occurrence of an event based on the proportion of screened predictors in the prediction model and derives the probability of occurrence of the relevant clinical event, assisting clinical staff in disease identification and management (26). It has great accuracy in forecasting the risk of disease incidence and can not only quantify one risk factor but also integrate many risk factors. Research on nomogram models for predicting the risk of social isolation among older adults has remained in the preliminary exploratory stage both domestically and internationally in recent years. To this day, Li et al. (27) are the sole researchers who have developed a nomogram prediction model for social isolation in older adults, utilizing the China Health and Retirement Longitudinal Study (CHARLS) database. Nevertheless, this study has its limitations, including its reliance on data from 2011, which may not align with current standards for assessing social isolation, potentially causing discrepancies with contemporary research. In the past decade, the widespread use of smartphones, the extensive adoption of social media platforms such as WeChat and Facebook, and the COVID-19 pandemic have markedly transformed the social dynamics of older adults. Consequently, the 2011 data fails to account for these new emerging risk or protective factors.

As a result, this study first identified risk factors that may affect social isolation in older adults, such as demographic characteristics, socioeconomic status, health factors, and health behavior factors, and then constructed and validated a risk prediction model for social isolation in older adults based on columnar plots, with the objective of effectively identifying and assessing those at high risk for social isolation in the older adult, predicting when social isolation might occur, and forecasting social isolation events. The model is created to function as a standard for the effective identification and screening of older adult adults who are at risk of social isolation, to predict the occurrence of social isolation in the older adult, and to provide healthcare professionals with timely preventive measures, even models to predict social isolation in older adult persons have not been established.

## 2 Methods

### 2.1 Study design

We identified a total of 18 variables associated or potentially connected to social isolation in four major categories based on pertinent research, group discussions, and expert consultation, and primarily collected the following information on older persons: (i) Demographic characteristics: gender, age, and marital status; (ii) Socioeconomic status: type of residence, income, and education; (iii) Health factors: BMI, health self-assessment, chronic disease, functional impairment (ADL), cognitive impairment, and depression; (iv) Health behaviors: current smoking/alcohol consumption, social activity, physical activity, nocturnal sleep duration, and daily sedentary duration.

Our sample size was calculated based on the formula (28) $n=[Z_{\alpha /2}^{2}P(1-P)]/\delta^{2}$ , when α=0.05 , $Z^{\alpha /2}=1.96$ , δ=0.05 ; according to the relevant literature, the incidence of social isolation among older adults was 31.4% (47). The sample size of the model was adopted as a 10-fold EPV rule of thumb (29) and there were 18 predictors of social isolation included in the pre-literature combing, at least 180 positive events were needed, then the training set should reach 581 cases. As internal data validation was adopted in this study, the ratio of training set to test set was 8:2 (30), and the total sample set should reach 726. Considering the 20% attrition rate, a minimum of 908 individuals were required for this survey.

### 2.2 Sampling strategy

We collected data using questionnaires mainly through two methods: door-to-door visits to households and gathering older adults at community activity centers. Six community nurses and seven graduate nursing students made up the survey team. All surveyors were given the same, standardized training. The survey was conducted one-on-one, face-to-face, with surveyors instructed to fill out the questionnaires and review them promptly, confirming missing items and filling them in addition, using uniform instructional language to explain the purpose of the study to the survey respondents. Respondents who are unable to complete the questionnaire on their own due to illness, low literacy, or other reasons will be assisted by surveyors, who will ask for clear and accurate answers to each item while avoiding subjective induced answers.

### 2.3 Participants

We conducted an older adult health survey in Hefei City, Anhui Province, China, from July to August 2022, using stratified cluster sampling. First we selected one representative jurisdiction within the four jurisdictions of Hefei city according to the random number method (Yaohai district was finally selected for the study). Next dividing Yaohai district into two strata based on urban and rural attributes, then using a simple random sampling method to draw one street or town in each stratum, and finally using a simple random sampling method to draw two communities or villages in each street or township as survey points, and selecting older adult adults aged 60 years or older as survey subjects. Inclusion criteria were as follows: (i) age of 60 years; (ii) clear consciousness and ability to complete the questionnaire; (iii) voluntary participation in this study and signing the informed consent form. Exclusion criteria: (i) Those with severe cognitive impairment or (and) mental disorder [MMSE <10 points or (and) GDS-15 > 9 points]; (ii) those with severe impairment of speech, vision, hearing, etc. Ultimately, 1,136 older adults were included in our study, which has exceeded the sample size that should have been collected in the formula and would have ensured an adequate sample. Internal data validation was used in this study, and these older adults were randomly divided into a training group (909 cases) and a validation group (227 cases) in an 8:2 ratio. The University’s Biomedical Ethics Committee gave its approval to this study (ID: 81220209), which was carried out in accordance with the Declaration of Helsinki.

### 2.4 Instrument

#### 2.4.1 Social isolation

To measure older adults’ social isolation, we used a condensed version of the Social Network Scale (LSNS-6) created by Lubben et al. (30). It has been validated for use in the Chinese older adult population and has shown good reliability and validity. This version is a simplified version of the original Social Network Scale created by Lubben, which uses brief questions to quickly and accurately assess the social network status of older adults (31). The scale includes six items, including family and friend isolation. A Likert 6-point scale was used, with scores ranging from 0 to 5, and a total score ranging from 0 to 30, with a total score of less than 12 indicating that the older person is socially isolated. Scores of less than 6 on the family isolation and friend isolation dimensions indicate that older adults are experiencing family and friend isolation. In this study, this scale’s Cronbach’s alpha coefficient was 0.774.

#### 2.4.2 Sociodemographic characteristics

The demographic information and socioeconomic state of the older adult are mostly covered in this section. Among them, demographic characteristics include age (years), gender (male or female), and marital status (married, unmarried, bereaved spouse, divorced). Socioeconomic status include type of residence (urban/rural), income (≤1,000 RMB, 1001–2000 RMB, 2001–3,000 RMB, 3001–5,000 RMB, ≥5,001 RMB), and education (primary and below, middle school, high school/junior college, university and above).

#### 2.4.3 Health factors

This section mainly collects the following information about the older adult: BMI (kg/m^2^), health self-assessment (good, better, general, rather poor), chronic disease (yes/no), functional impairment (ADL) (yes/no), cognitive impairment (yes/no), and depression (yes/no).

The specific scales used are as follows: (i) To measure functional impairment in older persons, we applied the Functional Activities of Daily Living Scale (ADL) created by Lawton and Brody (32), which has been validated and confirmed to have good reliability and validity in the Chinese older adult population (33). The scale includes 14 items, including the physical ability for daily living (PADL) and instrumental ability for daily living (IADL). A Likert 4-point scale was used, with scores ranging from 1 to 4, and a total score ranging from 14 to 56, where 14 indicates completely normal functioning and >14 indicates varying degrees of functional impairment. In this study, the scale’s Cronbach’s alpha coefficient was 0.904. (ii) To measure cognitive performance in older persons, we applied the Mini-Mental State Examination Scale (MMSE) created by Folstein et al. (34), which has been validated in a Chinese older adult population and has displayed great reliability and validity (35). Orientation, immediate memory, attention and calculation, recall, and language skills are among the 11 items on the scale. The total score is around 30, with higher total scores indicating better cognitive functioning. Mild cognitive impairment is defined as a total score of 21 to 26, moderate cognitive impairment as a score of 10 to 20, and severe cognitive impairment as a score of 0 to 9. In this study, the scale’s Cronbach’s alpha coefficient was 0.83. (iii) Using the Geriatric Depression Scale (GDS-15) created by Yesavage et al. (36), we evaluated depressed symptoms in older adult individuals. This scale has displayed great reliability and validity in the older adult Chinese population (37). It consists of 15 items with a “yes” and “no” question and answer scale with scores of 1 and 0, respectively, and a total score of 0 to 15. A total score of 0–4 is considered normal, 5–8 is considered mild depression, 8–11 is considered moderate depression, and 12–15 is considered severe depression. In this study, Cronbach’s alpha for the scale was 0.752.

#### 2.4.4 Health behavioral factors

This section mainly collects the following information about the older adult: current smoking (yes/no)/alcohol consumption (yes/no), social activity (frequently [≥3/week], some times [1-2/week], occasionally or never [≤1/week]), physical activity (daily, occasionally, never), nocturnal sleep duration (h), and daily sedentary duration (h).

### 2.5 Data analysis

**Figure 1:**
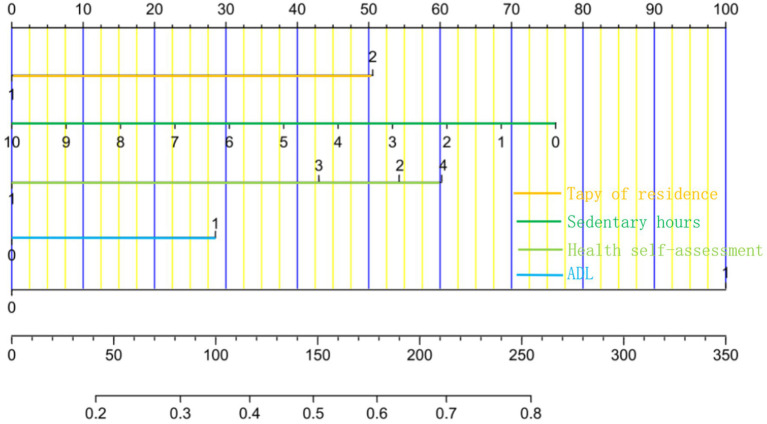
Nomogram prediction model for the risk of social isolation among the older adult in China. The variables for each horizontal line from top to bottom are type of residence, sedentary hours, health self-assessment, ADL, depression, total points, incidence of social isolation.

The data were analyzed and processed using SPSS 26.0 and R Studio. Using R Studio software, we then applied Mean ± Standard Deviation (x– ± s), frequency, and constituent ratio for statistical descriptions and *t*-tests or chi-square tests for group comparisons because our data information followed a normal distribution. The variables with *p*-value <0.05 in the univariate logistic regression analysis were included in the multivariate logistic regression model, and the backward stepwise regression method was applied to identify independent risk factors for the occurrence of social isolation in older adults. The nomogram consists of four components (Figure 1). An indicator score line, used to represent the risk level score of a single risk factor, is located at the top of the nomogram. Next, a risk factor line, used to represent the range of multiple potential risks for a single risk factor, is located below it. The number of risk factor lines is equal to the number of finalized risk factors. In the third place, there is a total score line, which represents the total risk score obtained by combining several risk variables; at the bottom of the nomogram, there is a predicted probability line that represents the likelihood that social isolation will occur based on the overall risk score.

Additionally, we validated the nomogram prediction model using the guidelines below: (i) the model’s external validation utilizing validation group data and internal validation using the Bootstrap approach with 1,000 repeated samples. (ii) Hosmer–Lemeshow and the area under the ROC curve were used to evaluate the nomogram model’s goodness of fit and discrimination (AUC). (iii) Calibration curves were created to evaluate the model’s precision. (iv) A Clinical Decision Curve Analysis (DCA) plot was created to evaluate the model’s clinical validity. In this research, differences were considered statistically significant at a *p*-value < 0.05.

## 3 Results

### 3.1 General information of the older adult

A total of 1,136 older persons were included. Of these, 478 older persons were in social isolation, an incidence of 42.1%.There were no statistically significant differences in demographic characteristics, socioeconomic status, health status, or health behaviors between the training and validation groups (*p*-value > 0.05).

### 3.2 Analysis of single factors affecting the occurrence of social isolation among older adults

The 909 study participants in the training group were grouped according to whether social isolation occurred for univariate analyses. As shown in Table 1, age in demographic characteristics, type of residence, and education in socioeconomic factors, BMI, health self-assessment, functional impairment, depression, and cognitive function in health status factors, and current smoking or not and sedentary hours in health behavior factors were all associated with the risk of social isolation in older adults (*p*-value < 0.05) (Table 2).

**Table 1:** Analysis of single factors affecting the occurrence of social isolation among older adults in China.

Variant	Social segregation group (*n* = 376)	Control subjects (*n* = 533)	*t/χ* ^2^	*P*
Demographic characteristics				
Age	70.85 ± 6.80	69.87 ± 6.45	−2.218	0.027
Gender			0.086	0.769
Male	150(39.9%)	213(40.0%)		
Female	226(60.1%)	320(60.0%)		
Marital status			2.922	0.404
Married	316(84.0%)	454(85.2%)		
Unmarried	1(0.3%)	0		
Literary	58(15.4%)	79(14.8%)		
Divorcee	1(0.3%)	0		
Socio-economic factors				
Type of residence			19.236	<0.001
Towns	297(79.0%)	477(89.5%)		
Urban	79(21.0%)	56(10.5%)		
Education			10.535	0.015
Primary and below	308(81.9%)	388(72.8%)		
Junior	36(9.6%)	83(15.6%)		
High school	26(6.9%)	50(9.4%)		
University and higher	6(1.6%)	12(2.3%)		
Monthly family income			5.705	0.222
≤1,000	227(60.4%)	291(54.6%)		
1,001–2000	63(16.8%)	91(17.1%)		
2001–3,000	41(10.9%)	64(12.0%)		
3,001–5,000	24(6.4%)	37(6.9%)		
≥5,001	21(5.6%)	50(9.4%)		
Health status factors				
BMI	24.10 ± 4.40	23.62 ± 2.82	−2.027	0.043
Chronic state			0.788	0.375
None	201(53.5%)	269(50.5%)		
Yes	175(46.5%)	264(49.5%)		
Health self-assessment			49.390	<0.001
Favorable	155(41.2%)	343(64.4%)		
Preferably	116(30.9%)	103(19.3%)		
Usual	86(22.9%)	77(14.4%)		
Mediocre	19(5.1%)	10(1.9%)		
Dysfunctional conditions			16.969	<0.001
None	273(72.6%)	447(83.9%)		
Yes	103(27.4%)	86(16.1%)		
Cognitive function			15.104	0.002
Normal	160(42.6%)	258(48.4%)		
Lightly damaged	167(44.4%)	244(45.8%)		
Moderately impaired	46(12.2%)	30(5.6%)		
Seriously damaged	3(0.8%)	1(0.2%)		
Depression			112.350	<0.001
None	168(44.7%)	420(78.8%)		
Yes	208(55.3%)	113(21.2%)		
Health behavior				
Smoking			5.227	0.022
None	350(93.1%)	472(88.6%)		
Yes	26(6.9%)	61(11.4%)		
Drinking			1.058	0.304
None	324(86.2%)	446(83.7%)		
Yes	52(13.8%)	87(16.3%)		
Duration of sleep	6.37 ± 1.39	6.53 ± 1.46	1.651	0.099
Sedentary hours	3.66 ± 2.41	4.17 ± 2.14	3.360	0.001
Social contact			3.358	0.187
Frequent	183(48.7%)	248(46.5%)		
Sometimes	59(15.7%)	109(20.5%)		
Occasionally or never	134(35.6%)	176(33.0%)		
Physical exercise			0.896	0.639
Daily exercise	251(66.8%)	368(69.0%)		
Sometimes	49(13.0%)	59(11.1%)		
Never	76(20.2%)	106(19.9%)		

**Table 2:** Assignment form of various factors influencing the occurrence of social isolation among the older adult in China.

Variant	Assign a value
Demographic characteristics	
Age (*X_1_*)	Original value entry
Socio-economic factors	
Type of residence (*X_2_*)	Towns = 0, Urban = 1
Education (*X_3_*)	Primary and below = *X_3_* (1), Junior = *X_3_* (2), High school = *X_3_* (3), University and higher = *X_3_* (4)
Health status factors	
BMI (*X_4_*)	Original Value Entry
Health self-assessment (*X_5_*)	Favorable = *X_5_* (1), Preferably = *X_5_* (2), Usual = *X_5_* (3), Mediocre = *X_5_* (4)
Cognitive impairment (*X_6_*)	None = 0, Yes = 1
Cognitive function (*X_7_*)	Normal = *X_7_* (1), Lightly damaged = *X_7_* (2), Moderately impaired = *X_7_* (3), Seriously damaged = *X_7_* (4)
Depression (*X_8_*)	None = 0, Yes = 1
Health behavior	
Smoking (*X_9_*)	None = 0, Yes = 1
Sedentary hours (*X_10_*)	Original Value Entry
Social isolation (*Y*)	None = 0, Yes = 1

### 3.3 Multi-factor analysis affecting the occurrence of social isolation among the older adult

The regression model was constructed using LR backward stepwise regression analysis, and the result indicates that type of residence, health self-assessment, functional impairment, depression, and sedentary hours were independent risk factors for the occurrence of social isolation among Chinese older adults, as shown in Table 3.

**Table 3:** Multifactorial logistic regression analyses influencing the occurrence of social isolation among Chinese older adults.

Variant	*β*	SD	Wald *χ*^2^	*P*	OR	95%CI
Socio-economic factors						
Type of residence (1)	0.657	0.212	9.635	0.002	1.929	1.274 ~ 2.921
Health status factors						
Health self-assessment			18.550	0.000		
Health self-assessment (1)	0.705	0.184	14.609	0.000	2.024	1.410 ~ 2.905
Health self-assessment (2)	0.559	0.200	7.818	0.005	1.749	1.182 ~ 2.588
Health self-assessment (3)	0.783	0.445	3.092	0.079	2.188	0.914 ~ 5.235
Cognitive impairment (1)	0.371	0.186	3.977	0.046	1.449	1.006 ~ 2.086
Depression (1)	1.300	0.158	67.346	0.000	3.668	2.689 ~ 5.003
Health behavior						
Sedentary hours	−0.099	0.034	8.721	0.003	0.906	0.848 ~ 0.967
Constant	−0.931	0.171	29.549	0.000	0.394	

### 3.4 Construction of a nomogram model of the risk of the occurrence of social isolation among the older adult

The logistic regression analysis’s independent risk factors for the occurrence of social isolation were incorporated into R Studio software to create a nomogram, as shown in Figure 1. The predicted probability of older adult social isolation is calculated by adding the scores of each variable and calculating the total score.

### 3.5 Validation of the nomogram prediction model

**Figure 2:**
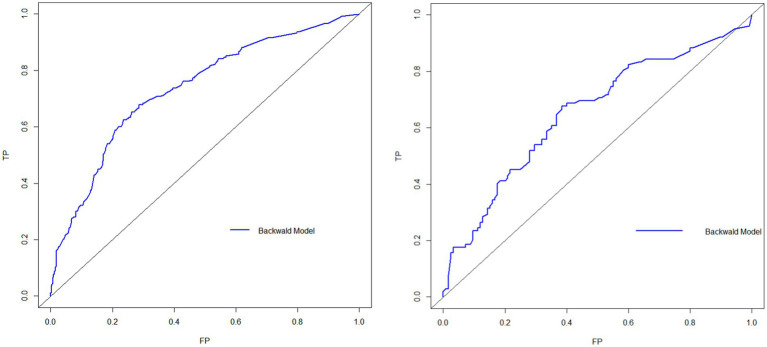
ROC curves for the occurrence of social isolation among older adults in China.

**Figure 3:**
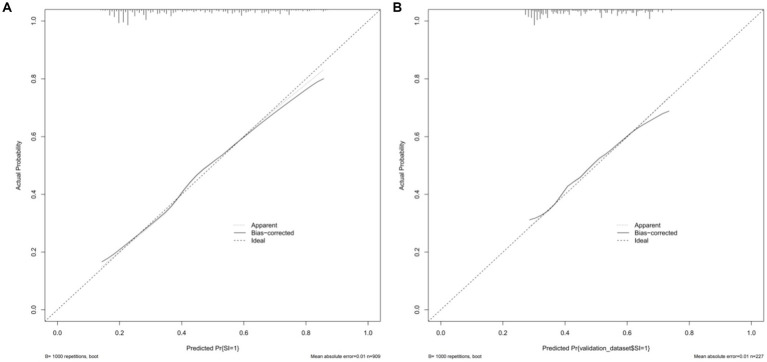
Prediction model calibration curves. **(A)** Internal validation calibration plot of the nomogram model predicting the risk of social isolation occurring in the older adult; **(B)** external validation calibration plot of the nomogram model predicting the risk of social isolation occurring in the older adult.

The model was validated in both the training and validation groups, with the AUC of the nomogram prediction model predicting the occurrence of social isolation in older adults in the training group being 0.734 (95%*CI*: 0.701 ~ 0.767) and the AUC of the nomogram prediction model predicting the occurrence of social isolation in older adults in the validation group being 0.653 (95%*CI*: 0.580 ~ 0.725), suggesting that the model discriminated better, as shown in Figure 2; Meanwhile, the Hosmer–Lemeshow test in the training group: *p*-value = 0.4044, demonstrated better goodness of fit, and the differences between its model fit curve and the ideal curve in both the training and validation group data were not statistically significant, indicating that the model predicted probabilities were generally consistent with the actual probabilities, as illustrated in Figure 3.

### 3.6 Clinical applicability of the nomogram prediction model

**Figure 4:**
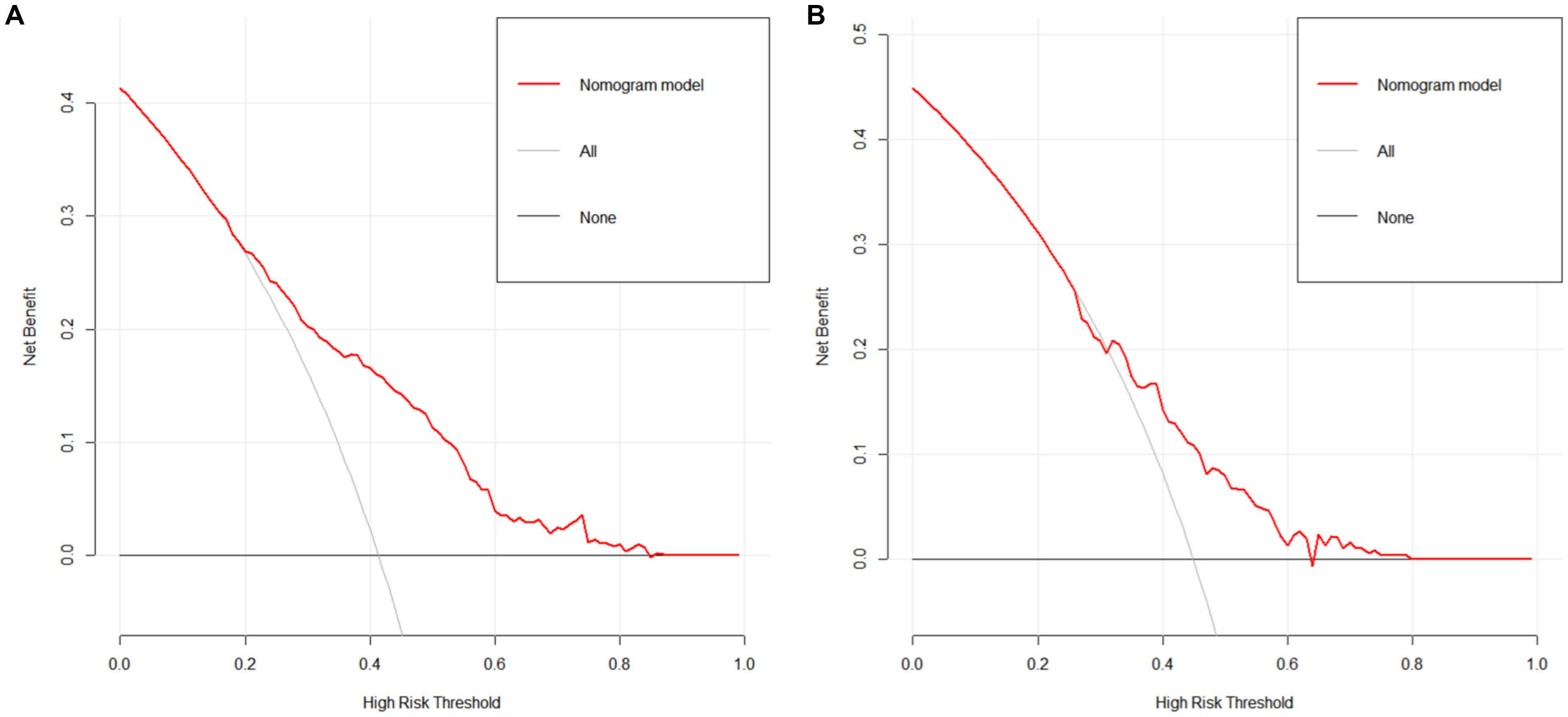
The prediction model’s DCA analysis. **(A)** DCA analysis of the training cohort line graph model predicting the risk of social isolation occurrence in the older adult; **(B)** DCA analysis of the validation cohort line graph model predicting the risk of social isolation occurrence in the older adult.

The clinical validity of the predictive model was evaluated using DCA, and Figure 4 depicts the DCA of the nomogram of the risk of social isolation in older adults. When the threshold probabilities for the training and validation groups were 20–85% and 30–65%, respectively, the results showed that clinical interventions for older adults had a high net benefit.

## 4 Discussion

In this study, we constructed and validated a simple nomogram model to predict the risk of social isolation in Chinese older adults considering five risk indicators, including the type of residence, sedentary time, health self-assessment, functional impairment, and depression. To ensure the model’s predictive accuracy, discrimination ability, and clinical utility, we created ROC curves, calibration curves, and DCA curves.

Being sedentary acts as a protective factor, whereas living situation, self-rated health, functional limitations, and depression contribute to the risk of social isolation. Specifically, first, older adults living in rural areas are more likely to be at risk of social isolation than those in urban areas. Rural residents commonly encounter inferior health outcomes due to a lack of public transportation, internet, and healthcare services (38, 39). In rural China, outdated community structures and recreational facilities restrict physical activity and social engagement for older adults. This increases the risk of social isolation for older adults living in the area. Our findings are in accordance with other research (40), however, the conclusion is also subject to some controversy. According to Henning-Smith et al. (39), urban residents are more experience social isolation compared to rural resident, but only for non-core rural older adults.

Secondly, similar to the results of the current study, Chatters et al. (18) found that older adults were more likely to experience social isolation if their self-rated health was worse. On one hand, older adult individuals who self-assess their health status as poor may not be able to actively participate in social activities due to a decline in physical functions, such as difficulty in mobility, reduced hearing, and vision. This leads to a decrease in their connection with society and increases the risk of social isolation. On the other hand, older adult people with a self-assessed poor health status may face more psychological stress, such as concerns about the deterioration of their condition and increased financial burdens. These stresses can lead to feelings of depression and a reduced willingness to socialize, further exacerbating social isolation. Lastly, older adult individuals with poor self-assessed health may be unable to go out due to physical reasons, reducing interactions with family, friends, and neighbors. This reduction in social activities may lead to less social support received, intensifying the degree of social isolation. When older adults start believing they are in good health, they are more likely to integrate and utilize social resources, improve their self-confidence in social participation and interpersonal communication, expand their social network, and reduce the risk of social isolation. As a result, for the older adult with poor self-assessment of health, we can provide rich geriatric education lectures and social activities to improve their health quality, encourage the older adult to do more health exercises, and maintain healthy eating behaviors, thereby reducing social isolation caused by poor self-assessment of health.

Thirdly, studies have shown that the ability to perform Activities of Daily Living (ADL) is an important predictor of the quality of life for the older adult (41). Older adult individuals with limited ADL capabilities often have a lower quality of life and are more prone to psychological issues such as depression and anxiety. In our study, impaired functioning of these self-care tasks increased the risk of social isolation in older adults. This is consistent with the findings of Na and Streim (20), who discovered that social isolation, particularly perceived isolation, increases with the stage of ADL in older adults. As they age, older persons experience declines in their physical and psychological health, which to varied degrees impacts their capacity to carry out daily tasks. Impaired functionality, a significant negative event in life that is directly related to health, can have a negative psychological impact on older persons. It can also limit their ability to actively participate in social interactions and passively reduce their social network, the risk of social isolation increasing. Preventing functional decline may aid in maintaining active social participation and lowering social isolation, as Na and Streim (20) propose. Additionally, Chatters et al. (18) indicated in their study that functionally impaired older adults may have a potentially beneficial impact on activating support from social networks and partaking in social interactions, especially reducing social isolation from friends, when they indicate the need for social care and help from others. As a result, we should focus on the changes in dysfunctional older adult social relationships and advocate for more family support and social care for dysfunctional older adult, such as organizing activities such as family fellowship, neighborhood mutual help, and peer support to assist dysfunctional older adult in maintaining and rebuilding their social contact network of family and friends, truly meeting their social and social needs, and creating a harmonious relationship network for dysfunctional older adult.

As reported in previous studies (1), Individuals suffering from depression frequently exhibit diminished interest and motivation in social activities, often actively reducing their interactions with others or even avoiding social gatherings. This social withdrawal is a significant aspect of depressive symptoms and a direct contributor to social isolation. Patients might opt to be alone because they feel inferior, helpless, or desperate, and believe that engaging in communication with others is challenging or devoid of meaning.

Sedentary times were an independent protective factor for social isolation in older adults, which was another significant finding. Older adults in the community spend the majority of their free time on static entertainment activities like watching TV, playing cards, chatting, and using computers or cell phones. The social isolation of older adult persons is not made worse by these static recreational activities, despite the possibility that passively increased sedentary time may result. Socially sedentary activities such as chatting with others and playing chess, for example, can help older adults make social connections (42). Sedentary behaviors such as Internet use by older adults can improve their social interactions and social connections (43). Although some studies have applied the time substitution hypothesis to explain that when people spend more time watching television or engaging in other sedentary activities, they spend less time engaging in other social activities as a result, weakening relationships and leading to social detachment (44). But for the time being, this conclusion is controversial because the older adult’s static recreational activities can also be viewed as a form of social interaction.

Currently, the common method used to assess social isolation is the Lubben Social Network Scale. According to Ge et al. (45) the Lubben Social Network Scale was used to categorize social isolation into four levels of risk. Additionally, some academics have created social isolation indices, such as the Berkman Social Network Index (46), to measure the prevalence of social isolation. These scales can evaluate isolation levels but lack the ability to predict social isolation risk. Second, scale scores and index scores may be insensitive to change, and one scale score or index by itself is insufficient to fully capture the complex nature of social isolation due to the regular exclusion of pertinent individual-specific indicators (such as demographics, health status) that are difficult to quantify. Instead, our nomogram model provides a novel alternative with a more intuitive and efficient approach. With ROC curve areas of 0.734 internally and 0.653 externally, the nomogram model exhibited strong predictive performance. It implies that the nomogram prediction model has good predictive and discriminatory abilities, and that it can better predict the occurrence of social isolation among the older adult in China. Aside from being simple to measure in community health management, the five risk factors included in the model are all individual demographic characteristics, physical-psychological health status, and health behaviors. These indicators better reflect the health status of older adults in multiple ways. The most vital point is that we note that the majority of earlier studies on social isolation risk factors (10, 18, 20, 21) used regression analytic methods to pinpoint individual risk factors affecting social isolation in older adults. As a result, these studies were limited in their ability to combine multiple disease risk factors to forecast the risk of a disease occurring. In this study, we constructed a nomogram model to identify multiple risk factors for social isolation in older adults and calculated a risk index to predict the risk of social isolation in older adults with multiple risk factors. Older adults living in rural areas with inactive lifestyles, negative health self-assessments, functional impairments, and depressive symptoms had a social isolation risk of more than 80%. We should focus on detecting and supervising older adults with multiple risk factors. Based on the findings of this study, we also need to construct an individual predictive model to recognize and screen early for groups at high risk of social isolation, particularly vulnerable populations like older persons in rural locations.

## 5 Conclusion

The study merges social cognition theory and a nomogram prediction model to examine social isolation among China’s older population. This identifies major influences on social isolation and provides a theoretical underpinning for future investigations into the social ties of seniors. The model is user-friendly and can help in evaluating and preventing social isolation among older adults.

The study, however, has its drawbacks. Although the model shows potential in identifying individuals at risk of social isolation, its predictive efficiency is limited. In the future, the generalization ability of models can be improved by collecting more data, using multi-center data to ensure that the data covers different scenarios and situations, thereby enhancing the predictive power of the models. It fails to examine the degree or forms of social isolation and is mainly centered on Chinese adults, limiting its generalizability to other demographics. As this is a cross-sectional study, it cannot establish causal relationships, and future longitudinal research is necessary. Moreover, validating the model with external data could enhance its applicability.

## Data Availability

The raw data supporting the conclusions of this article will be made available by the authors, without undue reservation.
